# Bazooka/PAR3 is dispensable for polarity in *Drosophila* follicular epithelial cells

**DOI:** 10.1242/bio.201410934

**Published:** 2015-03-13

**Authors:** Jaffer Shahab, Manu D. Tiwari, Mona Honemann-Capito, Michael P. Krahn, Andreas Wodarz

**Affiliations:** 1 Stammzellbiologie, Institut für Anatomie und Zellbiologie, Georg-August Universität Göttingen, Justus-von-Liebig-Weg 11, 37077 Göttingen, Germany; 2 Molekulare Zellbiologie, Institut I für Anatomie, Universität zu Köln, Kerpener Str. 62, 50937 Köln, Germany; 3 Cluster of Excellence – Cellular Stress Responses in Aging-associated Diseases, Joseph-Stelzmann-Str. 26, 50931 Köln, Germany; 4 Institut für Molekulare und Zelluläre Anatomie, Universität Regensburg, Universitätsstr. 31, 93053 Regensburg, Germany

**Keywords:** Polarity, Par complex, Epithelia

## Abstract

Apico-basal polarity is the defining characteristic of epithelial cells. In *Drosophila*, apical membrane identity is established and regulated through interactions between the highly conserved Par complex (Bazooka/Par3, atypical protein kinase C and Par6), and the Crumbs complex (Crumbs, Stardust and PATJ). It has been proposed that Bazooka operates at the top of a genetic hierarchy in the establishment and maintenance of apico-basal polarity. However, there is still ambiguity over the correct sequence of events and cross-talk with other pathways during this process. In this study, we reassess this issue by comparing the phenotypes of the commonly used *baz^4^* and *baz^815-8^* alleles with those of the so far uncharacterized *baz^XR11^* and *baz^EH747^* null alleles in different *Drosophila* epithelia. While all these *baz* alleles display identical phenotypes during embryonic epithelial development, we observe strong discrepancies in the severity and penetrance of polarity defects in the follicular epithelium: polarity is mostly normal in *baz^EH747^* and *baz^XR11^* while *baz^4^* and *baz^815^*^-8^ show loss of polarity, severe multilayering and loss of epithelial integrity throughout the clones. Further analysis reveals that the chromosomes carrying the *baz^4^* and *baz^815-8^* alleles may contain additional mutations that enhance the true *baz* loss-of-function phenotype in the follicular epithelium. This study clearly shows that Baz is dispensable for the regulation of polarity in the follicular epithelium, and that the requirement for key regulators of cell polarity is highly dependent on developmental context and cell type.

## Introduction

The defining characteristic of epithelial cells in all metazoans is apico-basal polarity. Its establishment and maintenance is essential for multiple cellular processes including cell-cell adhesion, cell-matrix adhesion, protein trafficking, cell shape and the regulation of growth and apoptosis. Given its vital role in cellular homeostasis, it comes as no surprise that defects in cell polarity can cause severe cellular disorders that have been directly linked to numerous diseases ranging from cancer to kidney dysfunction to blindness (Martin-Belmonte and Perez-Moreno, 2012; Tepass, 2012; Rodriguez-Boulan and Macara, 2014).

Apico-basal polarity in epithelial cells manifests upon the assembly of polarized cytoskeletal networks, polarized trafficking of vesicles and cargo and the localization of adhesion complexes to specific cortical locations. This causes division of the epithelial plasma membrane into distinct apical, basal, and lateral domains, typically enriched for specific phospholipid components and distinct, yet highly conserved, protein complexes (Johnson and Wodarz, 2003; McCaffrey and Macara, 2011; Tepass, 2012; Rodriguez-Boulan and Macara, 2014).

In *Drosophila* epithelial cells, the apical membrane is subdivided into a free apical membrane and a slightly basal subapical region (SAR) (Bilder et al., 2000; Tepass et al., 2001; St Johnston and Ahringer, 2010; Laprise and Tepass, 2011). The SAR is occupied by the Crumbs complex, composed of Crumbs (Crb), Stardust (Sdt), PATJ and Lin7, and the Par complex consisting of atypical protein kinase C (aPKC), Par6 and Bazooka/Par3 (Baz). These protein complexes are crucial for the establishment and maintenance of the apical plasma membrane domain (Bilder et al., 2003; Tanentzapf and Tepass, 2003; Harris and Tepass, 2008; Franz and Riechmann, 2010). The *Drosophila* basolateral plasma membrane is subdivided into the adherens junctions (AJs) or *zonula adherens* (ZA), the lateral membrane and the basal membrane. The AJs are key mediators of intercellular adhesion and lie just basal to the SAR. The core of the AJs is formed by the Cadherin-Catenin complex, composed of DE-cadherin (DE-cad), Armadillo/beta-catenin (Arm) and alpha-catenin. In addition, Baz as well as the immunoglobulin like adhesion molecule Echinoid (Ed) and its intracellular actin binding partner Canoe (Cno) localize to the AJs (Müller and Wieschaus, 1996; Wei et al., 2005; Harris and Tepass, 2010; Desai et al., 2013).

A second type of intercellular junction, the septate junction (SJ), also localizes to the lateral membrane. Associated with the SJ are the tumor suppressor proteins Lethal giant larvae (Lgl), Discs large (Dlg), Scribble (Scrib) and Fasciclin III (Bilder et al., 2000; Bilder et al., 2003; Tanentzapf and Tepass, 2003). The basal membrane is characterized by the localization of extracellular matrix receptors, namely integrins and Dystroglycan (Dg) (Tanentzapf et al., 2000; Schneider et al., 2006; Denef et al., 2008). Decades of research have revealed that these different regulatory protein complexes interact in an elaborate, yet highly conserved feedback loop to establish and maintain epithelial polarity (Bilder et al., 2003; St Johnston and Ahringer, 2010; Laprise and Tepass, 2011; Rodriguez-Boulan and Macara, 2014).

Epithelia in *Drosophila* can be distinguished into primary epithelia which derive from the embryonic blastoderm epithelium, and secondary epithelia which are generated by mesenchymal-epithelial transitions (Tepass et al., 2001). Epithelium formation in the *Drosophila* embryo occurs through a modified form of cytokinesis termed “cellularization”. Following fertilization, the *Drosophila* embryo undergoes 13 rounds of nuclear divisions without cytokinesis to form a syncytium comprised of roughly 6000 nuclei. Most of these nuclei align just below the embryonic surface where they become surrounded by plasma membrane invaginations to generate a uniform, highly polarized epithelium (Tepass et al., 2001; Harris, 2012; Choi et al., 2013). Several studies have shown that Baz plays a key role in the establishment and maintenance of apico-basal polarity during cellularization (Bilder et al., 2003; Harris and Peifer, 2004; Harris, 2012; Choi et al., 2013). At the onset of cellularization, localization of Baz to the apical circumference is mediated by Dynein and is mutually dependent on the actin-junctional linker, Cno. During cellularization, the localization and formation of AJs, as well as the apical localization of aPKC, Par6 (Harris and Peifer, 2005; Harris and Peifer, 2007) and Crb (Bilder et al., 2003) require Baz function. Baz is also crucial for zygotic epithelial development, as in its absence neuroectodermal cells lose apico-basal polarity, resulting in the formation of large holes in the ventral epidermis (Harris and Tepass, 2008).

While processes identical to cellularization have not been described in mammals, several similarities exist between the polarization of mammalian cells and secondary epithelia in *Drosophila* (Goldstein and Macara, 2007; St Johnston and Ahringer, 2010; Tepass, 2012; Rodriguez-Boulan and Macara, 2014). The follicular epithelium (FE) of *Drosophila* is an excellent example of a secondary epithelium. Somatic stem cells present in the germarium of the ovary divide asymmetrically to generate mesenchymal progenitors which generate FE cells via a mesenchymal-to-epithelial transition (Margolis and Spradling, 1995; Wu et al., 2008). Unlike the cellularizing embryo, which relies exclusively on apical cues for its polarization, the polarization of FE cells depends on apical, lateral and basal cues (Tanentzapf et al., 2000).

In spite of the phenotypic disparities between the developing FE and the cellularizing embryo, in-depth studies reveal stark similarities in the underlying molecular mechanisms and protein interactions regulating the polarization of both tissues (Tanentzapf et al., 2000; Franz and Riechmann, 2010; Morais-de-Sá et al., 2010; Rodriguez-Boulan and Macara, 2014). As in the cellularizing embryo, Baz plays a key role in the polarization of FE cells (Franz and Riechmann, 2010). During the early stages of polarization, Baz localizes to both the AJs and the apical membrane (Morais-de-Sá et al., 2010). At the AJs, it binds to Arm and Ed (Wei et al., 2005) and is required for their correct positioning (Franz and Riechmann, 2010; Morais-de-Sá et al., 2010). In the absence of Arm, Baz continues to localize to the cortex but extends ectopically into the basolateral domain and is distributed in clumps along the apical membrane, as opposed to forming a uniform belt (Franz and Riechmann, 2010). Thus, Baz and AJ components are mutually dependent on each other for positioning to their appropriate membrane domains.

Aside from these interactions, Baz also binds to Sdt, aPKC and Par6 and recruits them together with Crb to the apical domain as the development proceeds (Franz and Riechmann, 2010; Krahn et al., 2010b; Morais-de-Sá et al., 2010). Clonal analysis reveals that in early and late stage *baz* mutant FE clones, aPKC, Par6, Crb and Sdt fail to localize cortically (Franz and Riechmann, 2010; Morais-de-Sá et al., 2010). On the other hand, while apical localization of Baz is lost in early stage *crb*, *aPKC* and *par6* mutant clones, the AJ pool remains unaffected (Morais-de-Sá et al., 2010). Thus, according to the current model for establishment of apico-basal polarity in the FE, Baz functions at the top of a genetic hierarchy in the specification of the epithelial apical membrane (Franz and Riechmann, 2010).

As epithelia mature, apical Baz localization is gradually lost with the remaining protein localizing predominantly to the AJs while aPKC, Par6, Sdt and Crb colocalize at the SAR (Morais-de-Sá et al., 2010). Recent studies have shown that exclusion of Baz from the SAR is a key step in the establishment and maintenance of the apical membrane. Binding of Baz to aPKC and Sdt is inhibited by the phosphorylation of Baz by aPKC at Ser-980, while Crb outcompetes Baz for binding to Par6 (Morais-de-Sá et al., 2010; Walther and Pichaud, 2010; Krahn et al., 2010b; Laprise and Tepass, 2011). These dynamic and multifaceted interactions function together to facilitate the displacement of Baz from the SAR and promote its localization to the ZA (Harris and Peifer, 2005; McGill et al., 2009; Krahn et al., 2010b; Morais-de-Sá et al., 2010; Walther and Pichaud, 2010; Laprise and Tepass, 2011). The essential requirement for the exclusion of Baz from the SAR is reflected in the severe polarity defects caused by expression of a version of Baz that cannot be phosphorylated at Ser-980 and thus remains bound to aPKC and Sdt. FE cells expressing such a mutated version of Baz show persistent apical colocalization of Baz with aPKC, Par6, Crb and Sdt as well as relocalization of AJ proteins to the apical membrane (Krahn et al., 2010b; Morais-de-Sá et al., 2010).

Aside from its prominent role in the establishment and maintenance of apico-basal polarity in *Drosophila* epithelial cells, Baz plays key roles in various other cell types. It is required for the polarization of the developing oocyte and maintenance of oocyte cell fate (Huynh et al., 2001; Pinal et al., 2006; Becalska and Gavis, 2010). In neuroblasts, it functions in a complex with aPKC, Par6 and Inscuteable (Insc) to promote asymmetric cell division through the establishment of cortical polarity and regulation of spindle orientation (Wodarz et al., 1999; Schober et al., 1999; Wodarz et al., 2000; Petronczki and Knoblich, 2001; Rolls et al., 2003; Atwood et al., 2007; Hartenstein and Wodarz, 2013).

Most studies investigating the function of Baz in *Drosophila* development, including those characterizing its function in cellularization (Müller and Wieschaus, 1996; Bilder et al., 2003; Tanentzapf and Tepass, 2003; Harris and Peifer, 2004), zygotic epithelial development (Müller and Wieschaus, 1996; Bilder et al., 2003; Tanentzapf and Tepass, 2003; Harris and Tepass, 2008), embryonic neuroblasts (Schober et al., 1999; Wodarz et al., 1999; Atwood et al., 2007), oocyte polarity (Cox et al., 2001; Huynh et al., 2001; Doerflinger et al., 2010; Becalska and Gavis, 2010) and FE development (Abdelilah-Seyfried et al., 2003; Franz and Riechmann, 2010; Morais-de-Sá et al., 2010) have been conducted using the *baz^4^* (also known as *baz^xi106^*) and the *baz^815-8^* alleles. These alleles were considered null alleles, as they contain stop codons causing deletion of more than two thirds of the Baz protein (Krahn et al., 2010a).

In this study, we reassess the function of Baz in various developmental contexts using two previously generated yet phenotypically uncharacterized alleles, *baz^EH747^* and *baz^XR11^*. These two alleles are bona fide null alleles that do not give rise to any detectable Baz protein. We find that all *baz* alleles show similar defects in all developmental contexts assessed, except for in the FE. Here, *baz^EH747^* and *baz^XR11^* FE clones display normal apico-basal polarity unlike *baz^4^* and *baz^815-8^* clones, which frequently lose polarity and cause gaps in the epithelium. Our data indicates that the *baz^4^* and *baz^815-8^* chromosomes may carry additional mutations which strongly enhance the *baz* null phenotype. In contrast to previous reports, we show that Baz is not required for the correct positioning of AJs or the apical localization of aPKC, Par6, Sdt or Crb in the FE. Furthermore, apical plasma membrane domain formation also occurs normally in its absence. A genetic interaction screen reveals that the *baz* null FE phenotype is strongly enhanced by a reduction in Crb levels.

*baz^EH747^* and *baz^XR11^* FE clones show highly penetrant posterior follicular cell (PFC) multilayering, but the severity of multilayering is much milder than in clones for *baz^4^* and *baz^815-8^*. PFC multilayering is commonly observed in mutants affecting Hippo and Notch signaling, pointing to a potential involvement of Baz in these signaling pathways.

Overall, our results infer an auxiliary role for Baz in the polarization of FE cells and challenge the current model, which places it at the top of a genetic hierarchy in this process.

## MATERIALS AND METHODS

### Fly stocks and genetics

The following mutant alleles were used in this study: *baz^4^ FRT19A* (gift from Chris Doe), *baz^EH747^ FRT19A* (Eberl and Hilliker, 1988), *baz^XR11^ FRT19A* (Ralf Stanewsky, unpublished), *baz^815–8^ FRT19A* (McKim et al., 1996), *aPKC^K06403^ FRT42B* (Wodarz et al., 2000; Rolls et al., 2003), *crb^11A22^ FRT82B* (Tepass and Knust, 1993), *lgl^4^* (Hanratty, 1984), *par1^W3^ FRT42B* (Shulman et al., 2000), *scrib^2^ FRT82B* (Bilder et al., 2003), *PP2A^GE16781^* (GenExel) (Krahn et al., 2009), *PTEN^dj189^* (Gao et al., 2000), *shg^R69^ FRT42B* (Godt and Tepass, 1998), *FRT19A, Ubi-GFP FRT19A; e22c-gal4 UAS-Flp, e22c-gal4 UAS-Flp; FRT82B Ubi-GFP* (gift from Trudi Schupbach), *par6^Δ226^ FRT19A* and *cdc42^4^ FRT19A* (Jones and Metzstein, 2011), *ed^1x5^ FRT40A* (Wei et al., 2005), *cno^R2^ FRT82B* (Sawyer et al., 2009), *UAS-baz-GFP*, *UAS-baz-Δ312-1464-GFP*, *UAS-baz-Δ1-311-GFP* (Benton and St Johnston, 2003), *FRT19A tubP-Gal80LL1 hsFLP; tubP-gal4 UAS-mCD8-GFP* (gift from Heinrich Reichert), *yki-RNAi v104523* (Vienna Drosophila Resource Center), *baz-GFP-trap* CC01941 (Buszczak et al., 2007), *tj-GAL4* (Hayashi et al., 2002). The following stocks were obtained from the Bloomington Stock Center: *hsFlp^122^ FRT19A H2AvD-GFP* (#32045), *FRT19A* (#1709), *FRT19A ovoD* (#23880), *ovo-flp* (#8705), *GFP-RNAi* (#41556). The following lines were generated for this study: *baz^EH747^ FRT19A;;UAS-baz-GFP*, *baz^EH747^ FRT19A;;UAS-baz-Δ1-311-GFP*, *baz^EH747^ FRT19A;;UAS-baz-Δ312-1464-GFP*, *and baz^4^ FRT19A;;UAS-baz-GFP*.

### Generation of antibodies against Baz, Par6 and Lgl

An antibody directed against the three PDZ domains of Baz was generated by immunizing guinea pigs with a GST fusion protein containing amino acids 309–747 of Baz (Eurogentec, Seraing, Belgium). An antibody directed against Par6 was generated by immunizing rats with the peptide CHHQQAASNASTIMASDVKDGVLHL (Eurogentec, Seraing, Belgium). An antibody directed against Lgl was generated by immunizing guinea pigs with the peptides KGQQPSADRHRLQKDC and CNKIGTPKTAPEESQF (Eurogentec, Seraing, Belgium).

### Fixation of *Drosophila* embryos, larval brains and adult ovaries

Embryos were collected overnight at 25°C and dechorionated in bleach for 5 min. Embryos were washed with distilled water, transferred into a 1:1 mixture of heptane and 4% formaldehyde in PBS buffer and were then fixed by vigorous shaking for 30 min. Formaldehyde and PBS were removed and methanol was added to form a 1:2 mixture of heptane and methanol. Embryos were then devitellinized by vigorous shaking for 30 sec. After removing most of the liquid, devitellinized embryos were rinsed twice with methanol, incubated in methanol for 10 min and then rinsed twice with ethanol and stored at −20°C in ethanol for future use. For adult ovaries and larval brains, flies were reared at 25°C, dissected and fixed in Phosphate buffered saline (pH 7.4) (PBS), and fixed in 4% formaldehyde/PBS for 20 min. Fixed samples were washed 3 times in 0.1% Triton X-100/PBS (PBT) for 20 minutes and ovaries were then dissociated by pipetting up and down using a 1 ml pipette tip.

### Immunostaining of *Drosophila* embryos, larval brains and adult ovaries

Fixed ovaries or brains were blocked in 5% Normal Horse Serum (NHS)/1% Triton X-100/PBS for 30 min. Blocked ovaries and brains were rinsed 3 times in PBS to remove excess Triton X-100 and then incubated overnight at 4°C in 5% NHS/PBT with primary antibodies. Embryos were blocked in 5% NHS/PBT for 30 min and then incubated overnight at 4°C in 5% NHS/PBT with primary antibodies. Samples were washed 3 times for 20 min in PBT at room temperature. Samples were then blocked with 5% NHS/PBT for 30 min and incubated with secondary antibodies and DAPI in 5% NHS/PBT at 1:500 for 2 hours at room temperature. Secondary antibody solution was then removed and samples were washed 3 times for 20 min with PBT. Samples stained with fluorescent-conjugated secondary antibodies were mounted in Vectashield (Vector Laboratories, Burlingame, CA, USA) or Mowiol. Confocal microscopy was performed using a Zeiss LSM510 Meta confocal microscope. Images were processed on Photoshop CS3 (Adobe, San Jose, CA, USA) and assembled using Illustrator CS3 (Adobe, San Jose, CA, USA). The primary antibodies used were rabbit anti-Baz N-term (1:1000, Wodarz et al., 1999), guinea pig anti-Baz PDZ (1:1000), rat anti-Par6 (1:500), guinea pig anti-Mira (1:1000, Kim et al., 2009), guinea pig anti-Lgl (1:500), rat anti-Crb (1:500, gift from U. Tepass), mouse anti-Sdt (1:25, gift from E. Knust), rabbit anti-Cno (1:500, gift from M. Pfeifer), rabbit anti-lacZ (1:1000, MP Biosciences), rabbit anti-aPKC (1:500, Santa Cruz Biotechnology), mouse anti-GFP A11120 (1:1000, Molecular Probes), rabbit anti-GFP A11122 (1:1000, Molecular Probes). The following antibodies were obtained from the Developmental Studies Hybridoma Bank: rat anti-DE-cadherin DCAD2 (1:500), mouse anti-Dlg 4F3 (1:25), mouse anti-Orb 4H8 (1:20) and mouse anti-Armadillo N2 7A1 (1:50).

### Generation of FE clones

For negatively and positively marked FE clones generated under the control of *hsFlp*, animals of the appropriate genotype were heat shocked 5 days after egg laying (AEL) for 2 hours at 37°C on two consecutive days. Newly emerged flies were transferred to yeasted vials and allowed to mate for 4–5 days before dissection. To generate targeted mosaics in the FE, UAS-Flp was expressed under the control of e22c-Gal4 which is expressed in the somatic follicular stem cells. Newly emerged flies were transferred to yeasted vials and allowed to mate for 10 days before dissection. To generate maternal/zygotic clones, the dominant female sterile technique was used. When using hsFlp to drive recombination, animals of the appropriate genotype were heat shocked for 2 hours at 37°C 2 days in a row 1–2 days AEL and adult females of the desired genotype were collected. When using Ovo-Flp to drive recombination, flies of the appropriate genotype were crossed and adult females of the correct genotype were selected.

### SDS PAGE and western blot

Tissue was homogenized with a fitted pestle in a microcentrifuge tube in Tris-Sodium-Triton buffer (50 mM Tris pH 6.8, 150 mM NaCl, 1% Triton X-100) (TNT) and the concentration was quantified using the BCA Protein Assay Kit (Pierce, Thermo Fisher Scientific, MA, USA) according to the manufacturers instructions. Equal amounts of protein were diluted in 2× Laemmli Buffer (100 mM Tris pH 6.8, 200 mM DTT, 20% glycerol, 0.01% bromophenol blue and 4% SDS), boiled for 5 minutes, and subjected to electrophoresis in an SDS polyacrylamide gel for 60 min at 200 V using a BioRad electrophoresis apparatus. Proteins were transferred onto a nitrocellulose membrane for 60 min at 100 V; membranes were then blocked in 5% skim milk in TBST (Tris-buffered saline, 0.05% Tween-20) for 1 hour at room temperature and incubated with appropriate antibodies at 4°C overnight. Membranes were washed with TBST and incubated with appropriate horseradish peroxidase-labeled secondary antibody (Jackson ImmunoResearch, PA, USA) at a dilution of 1:5000 for 1 hour at room temperature. Membranes were then washed 3 times for 20 min in TBST. For chemiluminescent protein detection, membranes were incubated with BM Chemiluminescent substrate (Roche Diagnostics, Basel, Switzerland), exposed to photographic film and developed using Kodak developer. Blots were stripped by incubating them in stripping buffer (62.5 mM Tris-HCl pH 6.7, 100 mM 2-Mercaptoethanol, 2% SDS) at 60°C for 45 min. Blots were washed thoroughly in ddH_2_O, blocked in 5% skim milk in TBST for 1 hour and reprobed with appropriate antibodies.

### Data analysis

All data are reported as Mean ± S.D. of three independent experiments. Statistical analysis was conducted using R version 3.1.1; significance testing was performed with one-way ANOVA and post-hoc multiple testing was with Tukey's test.

## RESULTS

### FE phenotypes of *baz* mutant clones are allele-specific

**Fig. 1.**
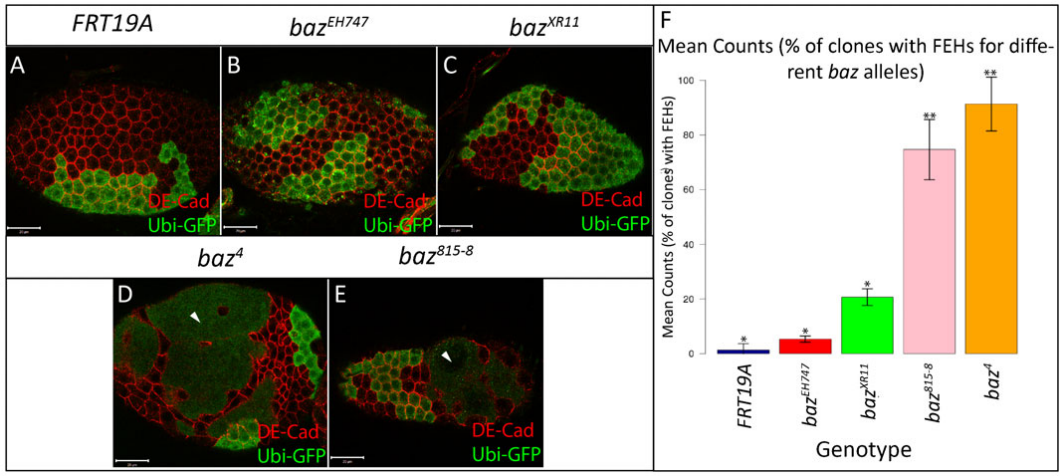
Different *baz* alleles display different FE phenotypes. FE clones marked by absence of GFP were generated using the directed mosaic system by expressing *UAS-Flp* under the control of *e22c-Gal4*. *FRT19A* (A), *baz^EH747^* (B) and *baz^XR11^* (C) mutant FE cells maintain a continuous epithelium while *baz^4^* (D) and *baz^815-8^* (E) mutant cells fail to maintain a continuous epithelium resulting in large holes in the FE, exposing the underlying nurse cells (white arrowheads). (F) Bar graph presenting quantitative analysis of the FEH phenotype. The number of *baz^4^*, *baz^815-8^*, *baz^EH747^*, *baz^XR11^* and *FRT19A* mosaic follicles with holes in the lateral epithelium were counted and presented as an average percentage from three repetitions of n = 50 (88% in *baz^4^*, 75% in *baz^815^*^-8^, 21% in *baz^XR11^*, 5% in *baz^EH747^*, 1% in *FRT19A*). At p<0.001, genotypes with same number of stars are not significantly different while those with different number of stars are significantly different. Error bars represent s.d. Scale bars = 20 µm.

**Fig. 2.**
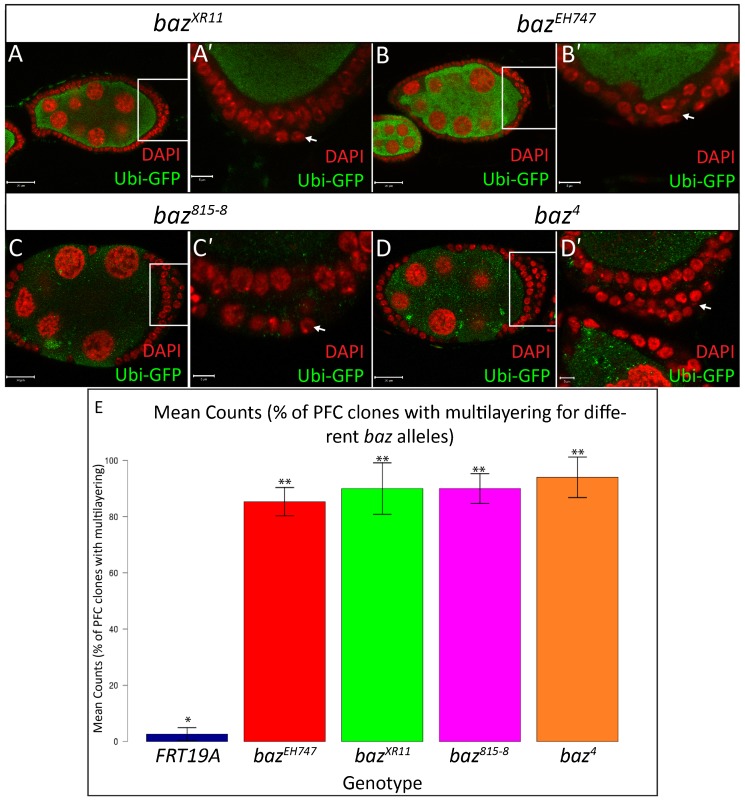
*baz* mutant posterior follicle cells show multilayering. Ovarian follicles in which *baz^XR11^* (A–A′, stage 7), *baz^EH747^* (B–B′, stage 7), *baz^815-8^* (C-C′, stage 8) and *baz^4^* (D–D′, stage 7) mutant cells marked by an absence of Ubi-GFP were generated using the directed mosaic system by expressing *UAS-Flp* under the control of *e22c-gal4*. White boxes in A,B,C,D indicate regions shown in A′,B′,C′,D′, respectively. Mosaic follicles were immunostained with GFP and stained with DAPI as indicated. PFCs mutant for *baz^XR11^* (A′), *baz^EH747^* (B′), *baz^815-8^* (C′) and *baz^4^* (D′) show multilayering as visualized by DAPI staining and indicated by arrows. (E) Bar graph presenting quantitative analysis of the PFC multilayering phenotype. The number of *baz^4^*, *baz^815-8^*, *baz^EH747^*, *baz^XR11^*, and *FRT19A* mosaic follicles with PFC multilayering were counted and presented as an average percentage from three repetitions of n = 50 (94% of *baz^4^*, 90% of *baz^815-8^*, 85% of *baz^XR11^*; 90% of *baz^EH747^*; 3% of *FRT19A*). At p<0.001, genotypes with same number of stars are not significantly different while those with different number of stars are significantly different. Error bars represent s.d. Scale bars = 20 µm (A–D); 5 µm (all other scale bars).

It was previously reported that large *baz* mutant clones positioned along the lateral FE lose integrity, which results in the formation of gaps and discontinuities, thus exposing the underlying nurse cells. On the other hand, *baz* PFC mutant clones were reported to show strong multilayering (Abdelilah-Seyfried et al., 2003). Our results confirmed these findings with regards to *baz^4^* and *baz^815^*^-8^ FE clones which often form large holes in the lateral FE and display strong multilayering of the PFCs (Figs 1 and 2). On the contrary, large holes or discontinuities were rarely observed in *baz^EH747^* and *baz^XR11^* lateral FE clones (Fig. 1), whereas careful examination of PFC clones revealed a mild, yet highly penetrant multilayering phenotype (Fig. 2). In contrast to PFC clones for *baz^4^* and *baz^815^*^-8^, which show severe multilayering throughout the clone, multilayering in *baz^EH747^* and *baz^XR11^* PFC clones is normally restricted to only a few cells (Fig. 2).

We quantitatively assessed the FE hole (FEH) phenotype by counting the number of *baz^4^*, *baz^815-8^*, *baz^EH747^*, *baz^XR11^* and FRT19A control mosaic follicles with lateral epithelial discontinuities. Our analysis revealed a distinct range in the penetrance of the FEH phenotype with *baz^4^* and *baz^815-8^* mosaic follicles showing far higher numbers of FEH compared to *baz^XR11^*, *baz^EH747^* and FRT19A control clones (Fig. 1; 88% in *baz^4^*, 75% in *baz^815^*^-8^, 21% in *baz^XR11^*, 5% in *baz^EH747^*, 1% in FRT19A. Percentages are an average from 3 repetitions with n = 50). Post-hoc multiple comparison tests showed that the differences in the penetrance of the FEH phenotype were significant between all genotypes tested albeit at a lower (95%) confidence level for *baz^EH747^* and *baz^XR11^* (Fig. 1F).

Quantitative analysis of the PFC multilayering phenotype revealed a high number of *baz^4^* (94%), *baz^815-8^* (90%), *baz^XR11^* (85%) and *baz^EH747^* (90%) multilayered PFC clones compared to the FRT19A control (3%). Post-hoc multiple comparison tests showed that the differences in the penetrance of the PFC multilayering phenotype in *baz^4^*, *baz^815-8^*, *baz^XR11^* and *baz^EH747^* clones were significant compared to FRT19A (Fig. 2).

**Fig. 3.**
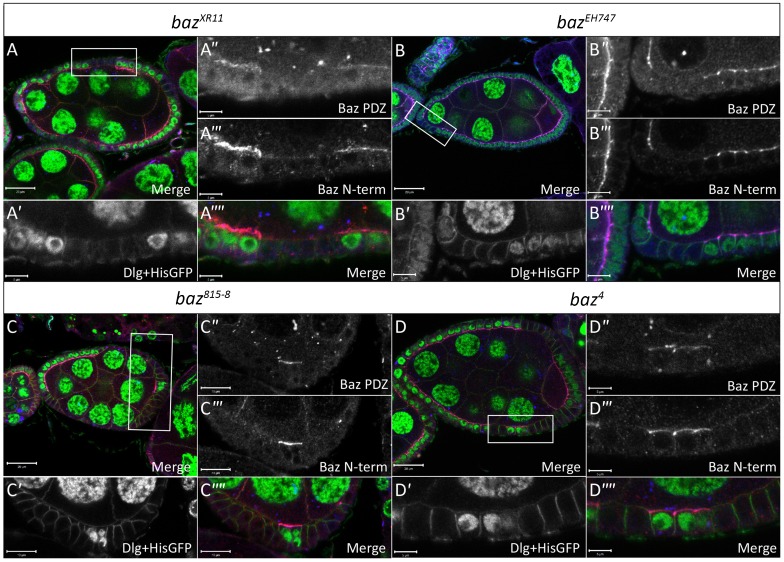
*baz* mutant cells lack Baz immunostaining. Ovarian follicles in which *baz^XR11^* (A–A″″), *baz^EH747^* (B–B″″), *baz^815-8^* (C–C″″) and *baz^4^* (D–D″″) mutant cells marked by loss of His-GFP were induced by *hs-Flp* mediated recombination. White boxes in A–D indicate regions shown in A′–A″″, B′–B″″, C′–C″″ and D–D″″, respectively. Mosaic follicles were immunostained with antibodies raised against amino acids 1-297 of the N-terminus of Baz (Baz N-term), amino acids 309-747 of the PDZ domain of Baz (Baz PDZ) and Dlg, which marks the basolateral cell boundaries as indicated. Both the Baz N-term and Baz PDZ antibodies show a complete absence of apical staining in *baz^XR11^* (A″ and A′″), *baz^EH747^* (B″–B′″), *baz^815-8^* (C″–C′″) and *baz^4^* (D″– D′″) mutant FE cells, respectively. Scale bars = 20 µm (A–D); 5 µm (all other scale bars).

To confirm that the FE defects observed in *baz^4^*, *baz^815-8^*, *baz^EH747^* and *baz^XR11^* clones were indeed due to an absence of Baz, we conducted immunostaining analyses with an antibody that was raised against the Baz N-terminal domain and another antibody against the Baz Postsynaptic density 95/Dlg/Zonula occludens 1 (PDZ) domains. With both antibodies Baz staining in *baz^4^*, *baz^815-8^*, *baz^EH747^* and *baz^XR11^* FE clones was below the limit of detection (Fig. 3). Furthermore, all FE defects observed in *baz^4^* (as previously reported by Benton and St Johnston, 2003; Morais-de-Sá et al., 2010) and *baz^EH747^* clones were rescued by overexpression of full length UAS-Baz using the MARCM system (supplementary material Fig. S1).

In order to obtain additional insight into which of the above mentioned alleles presented a genuine *baz* loss-of-function FE phenotype, we knocked down *baz* expression in FE cells by expressing *GFP-RNAi* in a *GFP-baz Trap* background under the control of *traffic jam-Gal4* (*tj-Gal4*) (supplementary material Fig. S2). Knockdown of *GFP-baz* was confirmed by GFP and Baz immunostaining (supplementary material Fig. S2B,B′). These *baz* deficient FE cells maintained a continuous epithelial sheet and displayed mild multilayering of the PFCs, similar to the phenotypes observed for *baz^EH747^* and *baz^XR11^* FE clones (supplementary material Fig. S2B,C″).

### *baz^4^* and *baz^815-8^* mutant animals express truncated Baz protein fragments

Given that we observed a significant difference in the penetrance of the FEH phenotype and severity of the PFC multilayering phenotype between different *baz* alleles, we sought to elucidate the underlying causes of these discrepancies. Differences in the penetrance of a given phenotype are commonly associated with the “strength” of the alleles in question. “Weak” hypomorphic alleles that retain some function often show relatively milder phenotypes compared to “strong” null alleles or deletion mutants. On the contrary, neomorphic mutations that result in the generation of a gene product with novel biological functions can show a more severe phenotype as compared to a null mutant of the same gene. Therefore, as a first step, we compared the individual characteristics of the aforementioned *baz* mutations.

**Fig. 4.**
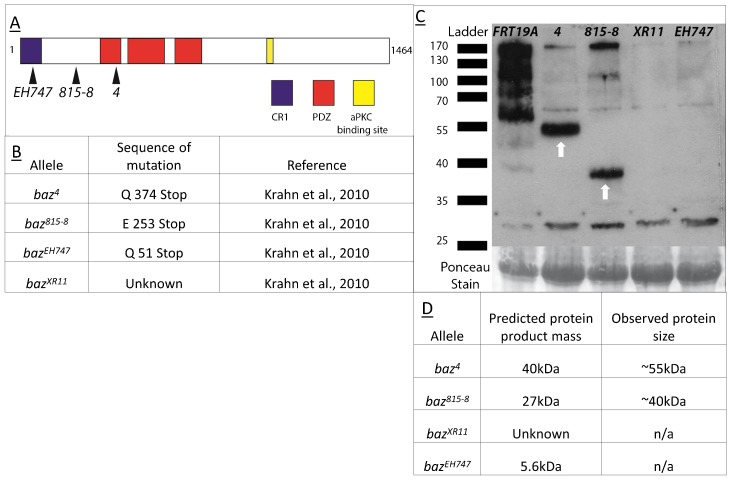
Schematic representation and sequence characteristics of *baz* mutant alleles. (A) Schematic representation of Baz protein with the CR1 domain shown in blue, PDZ domains in red and the aPKC binding site in yellow with black arrows indicating the approximate location of the stop codons present in the respective mutant alleles. (B) Table containing sequence details for the different *baz* mutant alleles. (C) Western Blot analysis of protein extracts from maternal zygotic *baz^4^*, *baz^815-8^*, *baz^XR11^*, *baz^EH747^*, and *FRT19A* mutant embryos. Bands at 55 and 40 kDa (white arrows) are observed in *baz^4^* and *baz^815-8^*. Faint bands are observed in all *baz* allele lanes at 170 kDa. (D) Summary of results of Western blot analysis of protein extracts from maternal zygotic *baz^4^*, *baz^815-8^*, *baz^XR11^*, *baz^EH747^* with a comparison to predicted protein product mass for each allele.

The *baz^4^* (Wieschaus et al., 1984) and *baz^EH747^* (Eberl and Hilliker, 1988) alleles were generated by Ethyl Methanesulfonate (EMS) mutagenesis, while the *baz^815-8^* (McKim et al., 1996) and *baz^XR11^* (Kuchinke et al., 1998; Ralf Stanewsky, unpublished) alleles were induced by X-ray mutagenesis (Fig. 4A,B). Based on sequencing data for the different *baz* alleles (Krahn et al., 2010a), we found that the position of the mutagenesis-induced stop codons in the *baz^4^*, *baz^815-8^* and *baz^EH747^* alleles predicts that each of these alleles should produce truncated Baz proteins of different lengths (Fig. 4B,D). The *baz^XR11^* allele does not contain any mutation in the coding region, pointing to a mutation in a regulatory element that prevents transcription or translation of the *baz* locus (Krahn et al., 2010a). Hypothetically, the *baz^4^* allele should generate a 40 kDa protein consisting of the first 374 amino acids of Baz, spanning the Conserved Region 1 (CR1) domain and a large portion of PDZ domain 1, while the *baz^815-8^* allele should produce a 27 kDa truncated protein consisting of the first 253 amino acids, also spanning the CR1 domain (Fig. 4A,B,D). The *baz^EH747^* allele should encode a 5.6 kDa, 51 amino acid fragment of the N-terminal region of Baz and should span only a portion of the CR1 domain (Fig. 4A,B,D).

To determine whether these truncated proteins affect the penetrance of the *baz* mutant FEH phenotype as well as the severity of PFC multilayering, we sought to establish whether they were indeed being stably expressed, or being post-translationally degraded. Western blot analysis of protein extracts from maternal zygotic (m/z) mutant embryos for the *baz^4^*, *baz^815-8^*, *baz^EH747^*, *baz^XR11^* and FRT19A as control revealed that truncated proteins were being stably expressed in the case of the *baz^4^* and *baz^815-8^* alleles, but not in case of the *baz^EH747^* and *baz^XR11^* alleles. Interestingly, these truncated proteins showed an upward shift in weight of approximately 15 kDa compared to the predicted sizes, which may be caused by some form of post-translational modification (Fig. 4C). Despite the detection of the truncated Baz proteins in Western blots of *baz^4^* and *baz^815-8^* embryos derived from germ line clones, we could not detect these Baz fragments by confocal microscopy, most likely because of their diffuse cytoplasmic distribution. This interpretation is supported by the diffuse cytoplasmic distribution of an overexpressed N-terminal fragment of Baz consisting of amino acids 1-311 (supplementary material Fig. S3; Benton and St Johnston, 2003).

### Expression of Baz N-terminal fragments does not interfere with FE integrity

The above data indicate a correlation between the presence of Baz N-terminal fragments with a minimum length of 253 amino acids and strong penetrance of the FEH phenotype as well as severe PFC multilayering. This could in turn lead one to speculate that *baz^4^* and *baz^815-8^* are neomorphic alleles and that the above mentioned Baz N-terminal fragments may be responsible for the high penetrance of the FEH phenotype and strong PFC multilayering, potentially through destabilization of epithelial polarity (Mizuno et al., 2003).

To assess these possibilities, we used the MARCM system (Lee and Luo, 2001) to express a *UAS-baz*Δ*312-1464-GFP* construct in *baz^EH747^* FE clones. As shown in supplementary material Fig. S3, there were no adverse effects on the integrity of the FE due to expression of a 311 amino acid N-terminal fragment of Baz. There was no statistical difference between the number of FEHs in *baz^EH747^*, *baz^EH747^;UAS-baz*Δ*312-1464-GFP*, and *FRT19A* mosaic follicles (supplementary material Fig. S3B). Furthermore, the severity of PFC multilayering was also not increased. These results indicate that the strong penetrance of the FEH phenotype in *baz^4^* and *baz^815-8^* mosaic follicles is unlikely to be due to the presence of Baz N-terminal fragments. This interpretation is supported by the fact that overexpression of *UAS-baz*Δ*312-1464-GFP* in a wild type background did not affect cell polarity or viability (data not shown; Benton and St Johnston, 2003).

### The *baz^EH747^* FE phenotype is enhanced by mutations for known polarity regulators

The above evidence indicated that *baz^4^* and *baz^815-8^* are not neomorphic alleles. An alternative explanation for the high penetrance of the FEH phenotype and strong PFC multilayering could be that second site mutations that enhance the *baz* mutant phenotype are present on the *baz^4^* and *baz^815-8^* chromosomes.

**Fig. 5.**
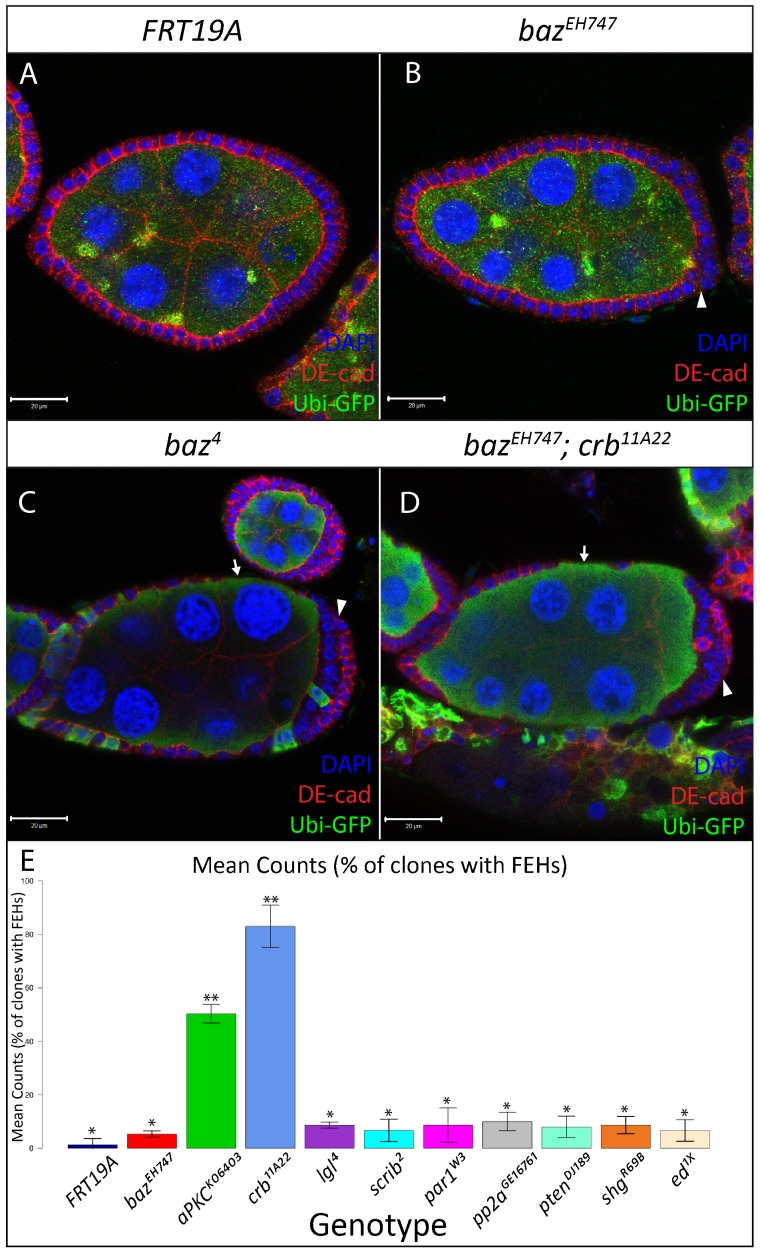
FE defects in *baz^EH747^* mosaic ovaries are enhanced by loss of one copy of *crb* or *aPKC.* FE clones for *FRT19A* (A), *baz^EH747^* (B), *baz^4^* (C), *baz^EH747^*; *crb^11A22^/+* (D), marked by absence of Ubi-GFP, were generated using the directed mosaic system by expressing *UAS*-*Flp* under the control of e22c-*gal4*. Lateral FE cells mutant for *baz^EH747^* (B) look identical to *FRT19A* (A) control epithelia and form a continuous epithelium over the underlying nurse cells. However, mild multilayering of the FE is visible at the posterior pole of *baz^EH747^* mosaic follicles (B, white arrowhead) as opposed to *FRT19A* control mosaic follicles (A). The *baz^EH747^* mutant phenotype is strongly enhanced in a *crb^11A22^* heterozygous mutant background (D) and appears identical to the *baz^4^* (C) FE mutant phenotype with discontinuities in the lateral FE (white arrows) and strong multilayering at the posterior poles (white arrowheads). (E) Bar graph presenting quantitative analysis of the FEH phenotype from the *baz* interaction screen. All clones analyzed are hemizygous mutant for *baz^EH747^* and heterozygous for the respective second mutation. The number of mosaic follicles with FEHs were counted and presented as an average percentage from 3 repetitions of n = 50 for each genotype listed (1% of *FRT19A*, 5% of *baz^EH747^*, 50% of *baz^EH747^;aPKC^K06403^*/*+*, 83% of *baz^EH747^;crb^11A22^*/*+*, 9% of *baz^EH747^;lgl^4^*/*+*, *7*% of *baz^EH747^;scrib^2^*/*+*, 9% of *baz^EH747^;par1^W3^*/*+*, 10% of *baz^EH747^;pp2a^GE16781^*/*+*, 8% of *baz^EH747^;pten^DJ189^*/*+*, 9% of *baz^EH747^; shg^R69B^*/*+*, 7% of *baz^EH747^;ed^1x5^*/*+*). At p<0.001, genotypes with same number of stars are not significantly different while those with different number of stars are significantly different. Error bars represent s.d. Scale bars = 20 µm.

We thus conducted a small-scale genetic interaction screen to test whether removing one copy of genes known to function in cell polarity enhances the *baz^EH747^* FEH and PFC multilayering phenotypes. Quantitative analysis of the results of this screen revealed a stark increase in the penetrance of the FEH phenotype and severity of PFC multilayering in *baz^EH747^* clones that are simultaneously heterozygous for *crb^11A22^* or *aPKC^k06403^* (Fig. 5). Post-hoc multiple comparison tests showed that differences in the penetrance of the FEH phenotype were significant between *baz^EH747^*; *crb^11A22^* and all other genotypes, and *baz^EH747^*; *aPKC^k06403^* and all other genotypes (Fig. 5). Intriguingly, *baz^EH747^* mosaic follicles heterozygous for *crb^11A22^* or *aPKC^k06403^* (data not shown) were phenotypically indistinguishable from *baz^4^* mosaic follicles (Fig. 5). These results raise the possibility that the chromosomes carrying the *baz^4^* and *baz^815-8^* alleles may carry second site mutations that synergistically enhance the *baz* loss-of-function phenotype.

### Comparison of the *baz^EH747^* and *baz^4^* phenotypes in other developmental contexts

Given that both *baz^EH747^* and *baz^4^* show distinct phenotypes in the FE, we next looked into whether these alleles show different phenotypes in other tissue specific contexts. We compared the cellularization phenotypes displayed by *baz^4^* and *baz^EH747^* maternal zygotic embryos. Previous studies using the *baz^4^* allele proposed that *baz* is epistatic to other members of the Par complex and the Crb complex during cellularization (Bilder et al., 2003; Harris and Peifer, 2004). Our analysis revealed that both *baz^4^* and *baz^EH747^* maternal zygotic mutants show similar phenotypes during cellularization, with aPKC and DE-cad failing to localize to their normal positions (supplementary material Fig. S4).

The *baz* gene was initially identified by its strong embryonic phenotype characterized by the formation of large holes in the ventral epidermis (Wieschaus et al., 1984; Müller and Wieschaus, 1996; Bilder et al., 2003; Harris and Tepass, 2008). Our comparison of zygotic epithelial development in *baz^4^* and *baz^EH747^* zygotic mutant embryos by immunostaining analysis revealed indistinguishable phenotypes characterized by large holes in the ventral epidermis in stage 13 embryos of both genotypes (supplementary material Fig. S5).

Baz has also been shown to function in oocyte development, where it is required for the oocyte to maintain its DNA in a haploid karyosome, in comparison to the 15 polyploid nurse cells with which it shares the egg chamber (Cox et al., 2001; Huynh et al., 2001). Furthermore, the oocyte fails to enrich Orb protein past stage 3 in the absence of Baz. Our comparison of the *baz^4^* and *baz^EH747^* alleles in this developmental context revealed identical defects during oocyte development with both *baz^4^* and *baz^EH747^* mutant oocyte nuclei becoming polyploid and failing to maintain Orb enrichment past stage 3 (supplementary material Fig. S6).

Lastly, we sought to compare the phenotypes of the *baz^4^* and *baz^EH747^* alleles in mitotic neuroblasts. We attempted to generate and compare *baz^4^* and *baz^EH747^* mutant neuroblasts in late stage 3rd instar larvae brains using the MARCM technique but were unable to identify *baz^4^* or *baz^EH747^* GFP marked larval mutant neuroblasts as compared to the FRT19A control (supplementary material Table S1). However, GFP marked neural progenitors were regularly identified in both *baz^4^* and *baz^EH747^* mosaic brains, raising the possibility that both *baz^4^* and *baz^EH747^* mutant neuroblasts are unable to maintain stem cell fate or undergo apoptosis.

Overall, the results of these experiments indicate that although the phenotypes for the *baz^4^* and *baz^EH747^* alleles differ dramatically in the FE, there are no discrepancies in cellularizing embryos, embryonic epithelia, oocytes or neuroblasts.

### Re-assessment of the function of Baz in FE cell polarity

As mentioned before, results from previous studies have led to the conclusion that Baz functions as a key regulator of cell polarity in *Drosophila* FE cells (Abdelilah-Seyfried et al., 2003; Franz and Riechmann, 2010; Morais-de-Sá et al., 2010). However, as the majority of these studies were conducted using the *baz^4^* and *baz^815-8^* alleles, whose strong FE phenotype differ from the true *baz* loss of function FE phenotype, the possibility arises that the true nature of the relationship between Baz and other polarity regulators may have been obscured. We thus reassessed these relationships by comparing the localization of components of the different polarity complexes in *baz^EH747^*, *baz^XR11^*, *baz^4^* and *baz^815-8^* lateral FE clones by immunostaining analysis.

**Fig. 6.**
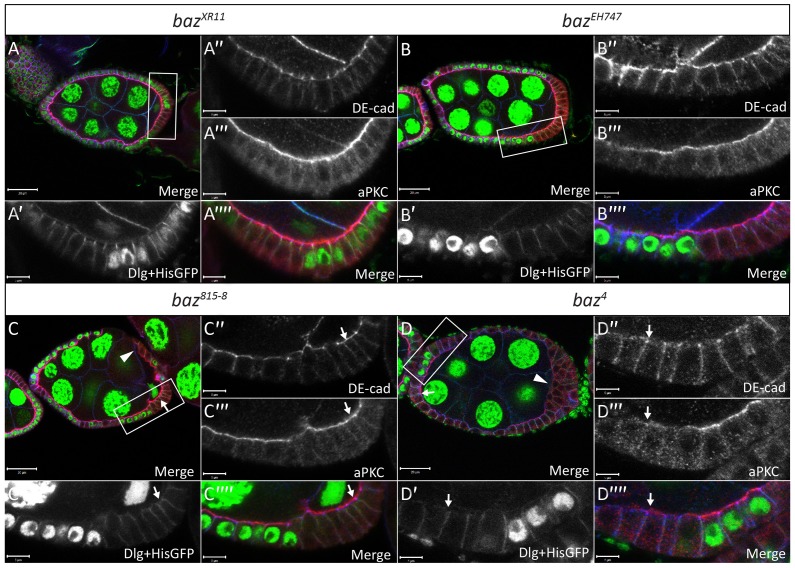
Localization of DE-cad and aPKC are unaffected in *baz^XR11^* and *baz^EH747^* FE clones. Ovarian follicles in which *baz^XR11^* (A–A″″), *baz^EH747^* (B–B″″), *baz^815-8^* (C–C″″) and *baz^4^* (D-D″″) mutant cells were induced by *hs*-*Flp* mediated recombination and marked by loss of His-GFP. White boxes in A,B,C,D indicate regions shown in A′–A″″, B′–B″″, C′–C″″ and D–D″″, respectively. Follicles were stained with DE-cad, aPKC and Dlg as indicated. Neither DE-cad nor aPKC apical localization is affected in *baz^XR11^* (A″ and A′″) or *baz^EH747^* (B″ and B′″) mutant cells. Many *baz^815-8^* mutant cells show wild type localization of aPKC and DE-cad as indicated by arrows (C–C″″). Some *baz^815-8^* mutant cells in which cell shape has been compromised appear to lose apical aPKC and junctional DE-cad staining as indicated by an arrowhead (C). Many *baz^4^* mutant FE cells retain junctional DE-cad localization even in the absence of apical aPKC localization as indicated by arrows (D–D″″). Others show a loss of both apical aPKC and junctional DE-cad as indicated by an arrowhead (D). Scale bars = 20 µm (A–D); 5 µm (all other scale bars).

We started by comparing the effects of the different alleles on the localization of aPKC and DE-cad in the lateral FE. Neither *baz^XR11^* (Fig. 6A–A″″) nor *baz^EH747^* (Fig. 6B–B″″) clones showed any difference in the localization of aPKC or DE-cad compared to neighboring wild type cells. Furthermore, *baz GFP RNAi* knockdown FE cells showed normal apical localization of aPKC and basolateral localization of Lgl (supplementary material Fig. S2C–C′″).

On the other hand, both *baz^815-8^* (Fig. 6C–C″″) and *baz^4^* FE (Fig. 6D–D″″) clones displayed a range of defects in the localization of aPKC and DE-cad as well as cell shape. In line with previous studies, *baz^4^* and *baz^815-8^* mutant lateral FE cells often displayed a flat cell shape with a stretched appearance (Fig. 6C, arrowhead) and showed a loss of apical aPKC and junctional DE-cad (Fig. 6C,D arrowheads) (Abdelilah-Seyfried et al., 2003; Morais de Sá et al., 2010). Additionally, we often observed intermediate phenotypes in *baz^815-8^* (data not shown) and *baz^4^* clones where mutant cells retained a relatively normal shape and displayed junctional DE-cad localization (Fig. 6D″, arrows) but strongly diminished apical aPKC localization (Fig. 6D′″, arrows). Furthermore, several *baz^815-8^* (Fig. 6C″,C′″) and *baz^4^* (data not shown) clones were present that displayed wild type cell shape with normal DE-cad and aPKC localization. Also, there were no *baz^815-8^* or *baz^4^* clones in which aPKC localized to the apical membrane but DE-cad failed to localize to the apical junctions.

**Fig. 7.**
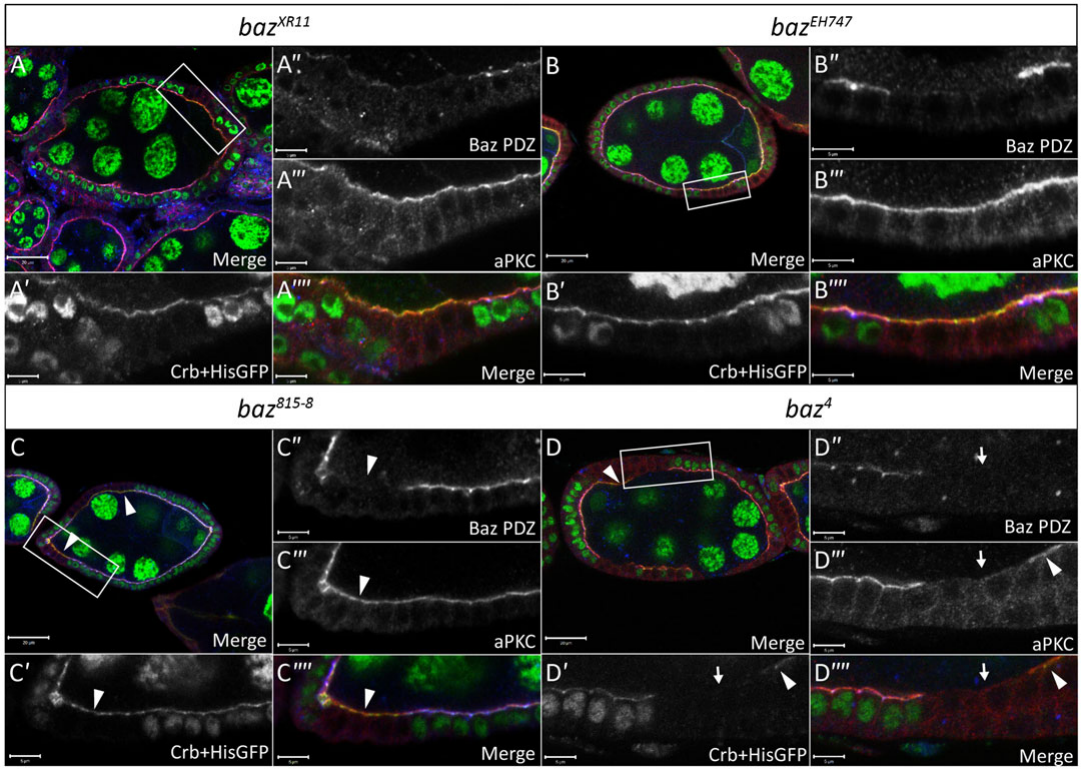
Localization of Crb is unaffected in *baz^XR11^* and *baz^EH747^* FE clones. Ovarian follicles in which *baz^XR11^* (A–A″″), *baz^EH747^* (B–B″″), *baz^815-8^* (C–C″″) and *baz^4^* (D–D″″) mutant cells were induced by *hs*-*Flp* mediated recombination and marked by loss of His-GFP and Baz immunostaining. White boxes in A,B,C,D indicate regions shown in A′–A″″, B′–B″″, C′–C″″ and D–D″″, respectively. Follicles were stained with anti-Baz-PDZ, aPKC and Crb antibodies as indicated. Baz is clearly absent from *baz^XR11^* (A″), *baz^EH747^* (B″), *baz^815-8^* (C″) and *baz^4^* (D″) mutant cells, respectively. No effect is seen on aPKC and Crb localization at the apical membrane in *baz^XR11^* (A′ and A′″) and *baz^EH747^* (B′ and B′″) mutant cells. Furthermore, no *baz^XR11^* (A) or *baz^EH747^* (B) mutant cells lose epithelial morphology. *baz^4^* (D′ and D′″) mutant cells show coincident loss of aPKC and Crb from the apical membrane as indicated by white arrows. However, apical aPKC and apical Crb staining is still visible in many *baz^815-8^* (C–C″″) and *baz^4^* (D–D″″) mutant cells as indicated by white arrowheads. Scale bars = 20 µm (in A–D); 5 µm (all other scale bars).

We next analyzed the effects of the different *baz* alleles on the localization of Crb. Our analysis of *baz^XR11^* and *baz^EH747^* FE clones revealed that loss of Baz had no effect on the localization of Crb (Fig. 7A,B″″). On the other hand, analysis of Crb localization in *baz^4^* and *baz^815-8^* FE clones yielded results similar to those for aPKC localization. In accordance with previous reports, Crb apical staining was often absent in *baz^4^* (Fig. 7D–D″″, arrows) and *baz^815-8^* (data not shown) mutant FE clones. However, we also observed many *baz^815-8^* and *baz^4^* mutant cells where Crb immunostaining appeared normal (Fig. 7C–C″″, arrowheads). Furthermore, unlike aPKC and DE-cad, aPKC and Crb localization appeared to be linked as all mutant cells that maintained apical Crb also maintained apical aPKC, while all those that showed a loss of apical Crb also showed a concomitant loss of aPKC.

**Fig. 8.**
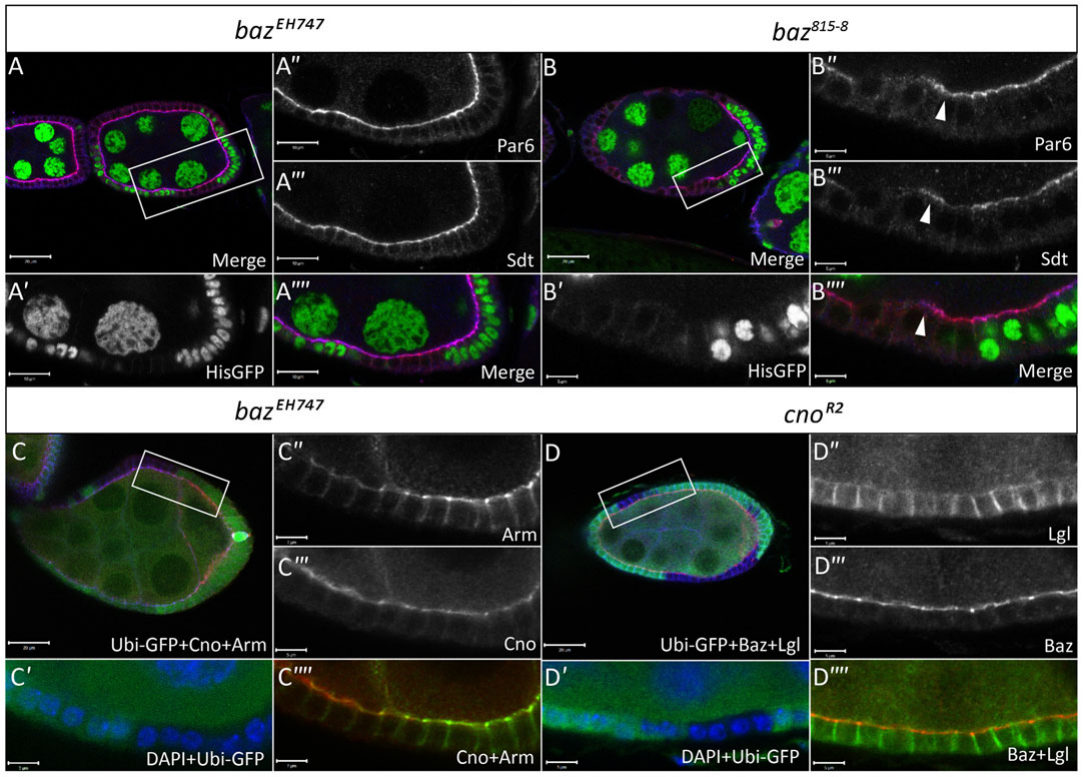
Baz and Cno do not regulate the localization of one another in the FE. Ovarian follicles in which, *baz^EH747^* (A–A″″, Stage 6) and *baz^815-8^* (B–B″″, Stage 8) clones induced by *hs-Flp* mediated recombination are marked by loss of His-GFP. White boxes in A,B indicate regions shown in A′–A″″ and B′–B″″, respectively. Follicles were stained with antibodies against Par6 and Sdt as indicated. No effect is seen on Par6 and Sdt localization at the apical membrane in *baz^EH747^* (A″,A′″) mutant cells. On the other hand, *baz^815-8^* (B″,B′″) mutant cells show coincident loss of Par6 and Sdt from the apical membrane. However, apical Par6 and Sdt staining is still visible in many *baz^815-8^* mutant cells (B″–B″″) as indicated by white arrowheads. (C–C″″) FE clones for *baz^EH747^* (stage 8) marked by absence of GFP and generated using the directed mosaic system by expressing UAS-Flp under the control of e22c-Gal4, immunostained with Cno, Arm and GFP antibodies as indicated. (D–D″″) *cno^R2^* clones (stage 7) immunostained with Baz, Lgl and GFP antibodies as indicated, marked by absence of GFP, were generated using the directed mosaic system by expressing UAS-Flp under the control of e22c-Gal4. White boxes in C and D indicate regions shown in C′–C″″ and D′–D″″, respectively. No effect is seen on Cno or Arm apical localization in *baz^EH747^* mutant FE clones (C–C″″) or Baz localization in *cno^R2^* mutant FE clones (D–D″″). Scale bars = 20 µm (A–D); 5 µm (all other scale bars).

We also analyzed the distribution of Par6 and Sdt, members of the Par complex and Crumbs complex, respectively, in *baz^EH747^* and *baz^815-8^* FE clones (Fig. 8A,B). Again, we observed no effect on the localization of these proteins in *baz^EH747^* FE clones. However, immunostaining for Par6 and Sdt in *baz^815-8^* FE clones (Fig. 8A,B) and *baz^4^* FE clones (data not shown) showed results similar to those obtained for aPKC and Crumbs.

A recent study has shown that during cellularization, Cno functions to localize Baz to the apical membrane and vice versa (Choi et al., 2013). However, up till now, no studies examining the relationship between Baz and the Ed/Cno complex in the FE have been reported. We thus examined Cno localization in *baz^EH747^* FE clones (Fig. 8C) and Baz localization in *cno^R2^* FE clones (Fig. 8D). No effect was seen on the apical localization of Cno in the absence of *baz* (Fig. 8C). FE clones for *cno* occasionally showed mild defects in FE development similar to the *baz^EH747^* phenotype (data not shown). However, the vast majority of *cno* clones showed no polarity defects and displayed normal Baz localization (Fig. 8D).

## DISCUSSION

### Discrepancies between *baz* alleles and elucidation of the true *baz* loss-of-function FE phenotype

Previous studies using the *baz^4^* and *baz^815-8^* alleles reported that *baz* is essential for polarization of the FE. *baz^4^* and *baz^815-8^* clones show large holes in the FE and strong multilayering of the PFCs. It was believed that *baz* functions at the top of a genetic hierarchy to specify the apical membrane as neither Par6, aPKC, Crb nor Sdt show apical localization in *baz^4^* and *baz^815-8^* mutant clones, whereas Baz cortical localization persists in *par6*, *aPKC*, *crb* and *sdt* mutant clones (Franz and Riechmann, 2010; Morais-de-Sá et al., 2010). On the contrary, our results from analyzing the *baz^EH747^* and *baz^XR11^* alleles indicate that *baz* does not play an essential role in polarization of the FE, as defects observed in *baz^EH747^* and *baz^XR11^* FE clones were statistically insignificant compared to *FRT19A* control clones. Furthermore, the localization of aPKC, Crb, Par6 and Sdt was unaffected in *baz^EH747^* FE clones. Following the observation of this discrepancy in phenotypes, we attempted to answer two important and interrelated questions. Firstly, which of these alleles represents the true *baz* loss-of-function phenotype and secondly, what is the underlying cause of the differences observed between these alleles.

Our immunostaining analysis revealed that Baz protein was absent from FE clones for all *baz* alleles examined. This immunostaining data was further corroborated by our western blot analysis, which revealed the absence of Baz protein in *baz^EH747^* and *baz^XR11^* and the presence of truncated Baz proteins in *baz^4^* and *baz^815-8^* protein extracts. Both the mild multilayering phenotype of the *baz^EH747^* and *baz^XR11^* alleles as well as the strong multilayering phenotype and FEHs of the *baz^4^* and *baz^815-8^* alleles can be rescued by overexpression of *UAS-Baz-GFP*, indicating that a loss of full-length Baz underlies both the strong and mild FE phenotypes. Based on these results, it can be postulated that the *baz^4^* and *baz^815-8^* lines carry enhancer mutations or that they function as neomorphic alleles due to the expression of truncated Baz protein fragments. Alternatively, the *baz^XR11^* and *baz^EH747^* lines may carry additional suppressor mutations. However, the phenotype generated by *GFP-RNAi* directed against *baz-GFP Trap*, which occurs in the absence of truncated Baz protein fragments or any potential enhancer or suppressor mutations present in the aforementioned lines, strongly resembles the *baz^EH747^* and *baz^XR11^* phenotype. Thus, these data argue in favor of *baz^XR11^* and *baz^EH747^* representing the true *baz* loss-of-function phenotype in the FE.

We next sought to determine whether the underlying cause of the severe phenotype displayed by the *baz^4^* and *baz^815-8^* alleles is due to the production of truncated Baz protein fragments that interfere with polarity or the presence of additional enhancer mutations in these lines. Expression of a Baz N-terminal fragment in a *baz^EH747^* mutant background failed to enhance the mild *baz* loss-of-function phenotype, indicating that *baz^4^* and *baz^815-8^* are unlikely to be neomorphic alleles. On the other hand, removal of one copy of *crb* or *aPKC* from *baz^EH747^* FE clones resulted in severe defects that phenocopy *baz^4^* and *baz^815-8^* FE clones. These data provide strong evidence that the strains carrying the *baz^4^* and *baz^815-8^* alleles contain additional mutations that enhance the true *baz* loss-of-function phenotype. As the *baz^4^* and *baz^815-8^* alleles were generated by EMS and X-ray mutagenesis respectively, it appears likely that the respective chromosomes accumulated additional mutations. However, given that the FE defects observed in *baz^4^* clones can be fully rescued by expression of full length Baz (supplementary material Fig. S1; Benton and St Johnston, 2003; Morais-de-Sá et al., 2010), the high penetrance of the FEH phenotype and severe PFC multilayering is likely to be the result of a synergistic enhancement of the *baz* mutant phenotype by additional mutations which do not result in FE defects on their own. Given that *sdt* and *par6*, two known *baz* interactors are also located on the first chromosome, one could speculate that hypomorphic mutations in either of these genes could result in the strong penetrance of FE phenotypes observed in *baz^4^* and *baz^815-8^* clones.

### Baz is required for establishment of polarity during cellularization, but is dispensable for polarity in the FE

The vast majority of *baz^EH747^* and *baz^XR11^* FE clones showed normal polarization of the FE and maintained aPKC, Par6, Crb and Sdt localization. On the other hand, *baz^EH747^* m/z mutant embryos were unable to establish apico-basal polarity during cellularization, reflected in their failure to localize DE-cad and aPKC to their appropriate locations. From these results, it can be concluded that *baz* plays an essential role in the establishment and maintenance of polarity during cellularization, but not in the FE. This raises the important question as to why the requirement for *baz* function differs in these two developmental contexts.

One explanation for this discrepancy could be that Baz functions redundantly with another protein in the establishment and maintenance of apico-basal polarity in the FE as compared to the embryo. Several lines of evidence indicate that *crb*, which also displays severe defects in the apico-basal polarity in the embryo, but mild defects in the FE, may function redundantly with *baz* in certain developmental contexts (Morais-de-Sá et al., 2010; Walther and Pichaud, 2010; Fletcher et al., 2012; Thompson et al., 2013). For example, during FE development, Baz initially colocalizes apically with aPKC and Par6 but is gradually replaced by Crb and restricted basally to the AJs (Franz and Riechmann, 2010), indicating that Crb can function redundantly with Baz in the maintenance of polarity. Our experiments revealed that the *baz^EH747^* FE phenotype is strongly enhanced by the loss of one copy of *crb* while a previous report from Morais-de-Sá et al., 2010. (Morais-de-Sá et al., 2010) showed that overexpression of Baz can rescue *crb* mutant FE defects. These data taken together could lead one to speculate that Crb and Baz can substitute for each other in the establishment and maintenance of apico-basal polarity in the FE. However, Crb expression and localization to the apical membrane of FE cells occurs at stage 1, sometime after Baz, aPKC and Par6 are detectable in region 2B of the germarium (Franz and Riechmann, 2010). Thus, Crb is unlikely to substitute for Baz in establishing polarity in the FE.

Another potential explanation for the divergent requirements for *baz* in the establishment of epithelial polarity during cellularization vs FE development is the difference in the underlying polarity cues operating in these tissues. As mentioned earlier, FE cells are derived from somatic stem cells in the ovarian niche and make use of basal, lateral and apical cues to establish polarity as they undergo a mesenchymal-epithelial transition. For example, contact between polarizing FE precursors and the underlying basal lamina is sufficient for the specification of the basal membrane, while the germ cells act as a signal for polarization and specification of the apical and lateral membranes (Tanentzapf et al., 2000). On the other hand, the only polarization signal that has been identified during cellularization is the embryonic membrane, which goes on to form the apical membrane once cellularization is complete (Mavrakis et al., 2009; Choi et al., 2013). Therefore, one could speculate that the presence of multiple polarity cues in the FE is sufficient to compensate for an absence of *baz* during the establishment of polarity. This explanation is further supported by our finding that Cno, which is essential for establishing apico-basal polarity in the cellularizing embryo, displayed only mild polarity defects in the FE, similar to *baz*. Thus, it can be hypothesized that Baz function in the establishment of FE polarity may be redundant with other basal, lateral and apical polarity cues, while its function in the maintenance of apico-basal polarity could be redundant with Crb.

### A potential function for *baz* in Hippo and Notch signaling

PFC multilayering is a trademark phenotype of several genes involved in Hippo signaling and results from unrestricted cell division (Meignin et al., 2007; Polesello and Tapon, 2007; Genevet et al., 2010; Yue et al., 2012). The high penetrance of this multilayering phenotype in *baz* PFC clones raises the possibility that Baz too may function in Hippo signaling. Several studies have provided strong evidence showing that known interactors of Baz, namely Crb and aPKC, interact with members of the Hippo signaling pathway to regulate cell division and tissue growth (Chen et al., 2010; Ling et al., 2010; Parsons et al., 2010; Robinson et al., 2010). Thus, it comes as no surprise that the activities of Baz may also impinge on this pathway, either through direct interaction with core Hippo pathway members or indirectly through the regulation of aPKC and Crb in their function as apico-basal polarity regulators.

Regulation of PFC division by Hippo signaling occurs through the Notch pathway (Maitra et al., 2006; Polesello and Tapon, 2007; Yu et al., 2008). Notch is required for the mitotic to endocycle switch at stage 6 by upregulating the zinc finger transcription factor *hindsight*, which functions to downregulate the homeodomain gene *cut* (Sun and Deng, 2005; Sun and Deng, 2007). Mislocalization of Notch through disruption of apical basal polarity or defective vesicle sorting has been shown to result in excess PFC proliferation and multilayering (Deng et al., 2001; Yan et al., 2009). Thus, one could speculate that the efficacy of Notch localization and signaling may be attenuated in a *baz* mutant background, where the systems regulating polarity are highly sensitized, therefore resulting in mild PFC multilayering. Further studies are required to determine how exactly *baz* functions to restrict PFC proliferation and whether it mediates its effects through Hippo, Notch or other pathways.

## CONCLUSIONS

In conclusion, the results of this study provide evidence that chromosomes carrying the commonly used *baz^4^* and *baz^815-8^* alleles may carry additional mutations that enhance the true *baz* loss-of-function phenotype in the FE. Our results show that contrary to previous studies using the aforementioned alleles, *baz* is not essential for the establishment and maintenance of FE cell polarity. Our analyses of the true *baz* loss-of-function phenotype in the FE indicate that *baz* does not function at the top of a genetic hierarchy in the localization of apical membrane determinants such as Crb and aPKC or AJ components such as DE-cad or β-catenin. This study provides an intriguing example of how the function and requirement for regulatory proteins and their corresponding signaling pathways can vary drastically depending on the developmental context and cell type.

## Supplementary Material

Supplementary Material
