# Supplementary material for: Bazooka/PAR3 is dispensable for polarity in Drosophila follicular epithelial cells
Source: Biol Open. 2015 Mar 13;4(4):528–41. doi: 10.1242/bio.201410934 (PMC4400595; doi:10.1242/bio.201410934)
Supplement: Supplementary Material [file supp_4_4_528__index.html]

Bazooka/PAR3 is dispensable for polarity in Drosophila follicular epithelial cells — Bazooka/PAR3 is dispensable for polarity in Drosophila follicular epithelial cells — Supplementary Material 

# Bazooka/PAR3 is dispensable for polarity in *Drosophila* follicular epithelial cells

## bio.201410934 Supplementary Material

**Files in this Data Supplement:**

- Supplementary Material - Jaffer Shahab et al. doi: 10.1242/bio.201410934
