# Supplementary material for: Bazooka/PAR3 is dispensable for polarity in Drosophila follicular epithelial cells
Source: Biol Open. 2015 Mar 13;4(4):528–41. doi: 10.1242/bio.201410934 (PMC4400595; doi:10.1242/bio.201410934)
Supplement: Supplementary Material [file supp_bio.201410934_bio.201410934-s1.pdf]

Biology Open (2015) 000, 1–14 doi:10.1242/bio.201410934

Supplementary Material

Jaffer Shahab et al. doi: 10.1242/bio.201410934

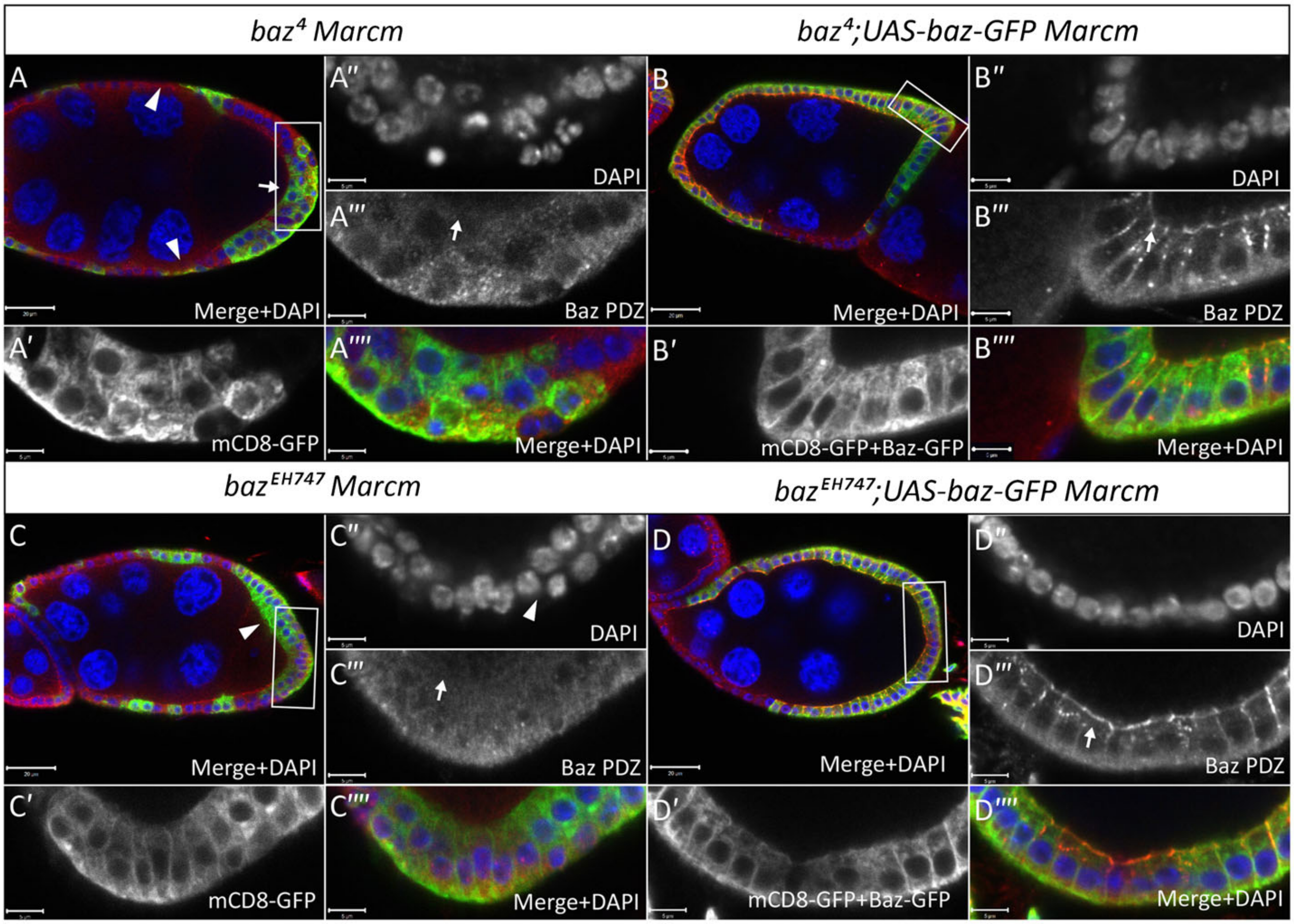

Fig. S1. FE defects in *baz* mutant clones are rescued by expression of UAS-Baz-GFP. Mosaic ovarian follicles in which *baz*^{4} (A–A⁗), *baz*^{4}; *UAS-baz-GFP* (B–B⁗) *baz*^{EH747} (C-C⁗), and *baz*^{EH747}*;UAS-baz-GFP* (D–D⁗) mutant follicle cells were induced using the MARCM technique by *hs-Flp* mediated recombination and marked by expression of mCD8-GFP. White boxes in A–D indicate regions shown in A′–A⁗, B′–B⁗, C′–C⁗ and D′–D⁗, respectively. Follicles were immunostained with antibodies raised against the PDZ domain of Baz (Baz PDZ) and GFP and stained with DAPI as indicated. Discontinuities in the lateral FE (A, white arrowheads) and multilayering of cells at the posterior pole (A, white arrow and A′–A⁗) are observed in *baz*^{4} mosaic follicles. These defects are not observed in *baz*^{4}; *UAS-baz-GFP* mosaic follicles where a continuous FE layer covers the follicle (B) and no multilayering is seen at the posterior pole (B′–B⁗). Multilayering of the posterior follicle cells is observed in *baz*^{EH747} (C and C″ as indicated by white arrowhead). These defects are not observed in *baz*^{EH747}*;UAS-baz-GFP* (D–D⁗) mosaic follicles where the PFCs form a uniform monolayer covering the underlying oocyte. In *baz*^{4}; *UAS-baz-GFP* and *baz*^{EH747}*;UAS-baz-GFP* mosaic follicles, apical localization of UAS-Baz-GFP is clearly visualized by staining with the Baz-PDZ domain antibody (B‴ and D‴, white arrow). This apical localization is absent in *baz*^{4} and *baz*^{EH747} mutant cells (A‴ and C‴, respectively, white arrow). Scale bars=20 µm (A–D); 5 µm (all other scale bars).

Biology Open (2015) 000, 1–14 doi:10.1242/bio.201410934

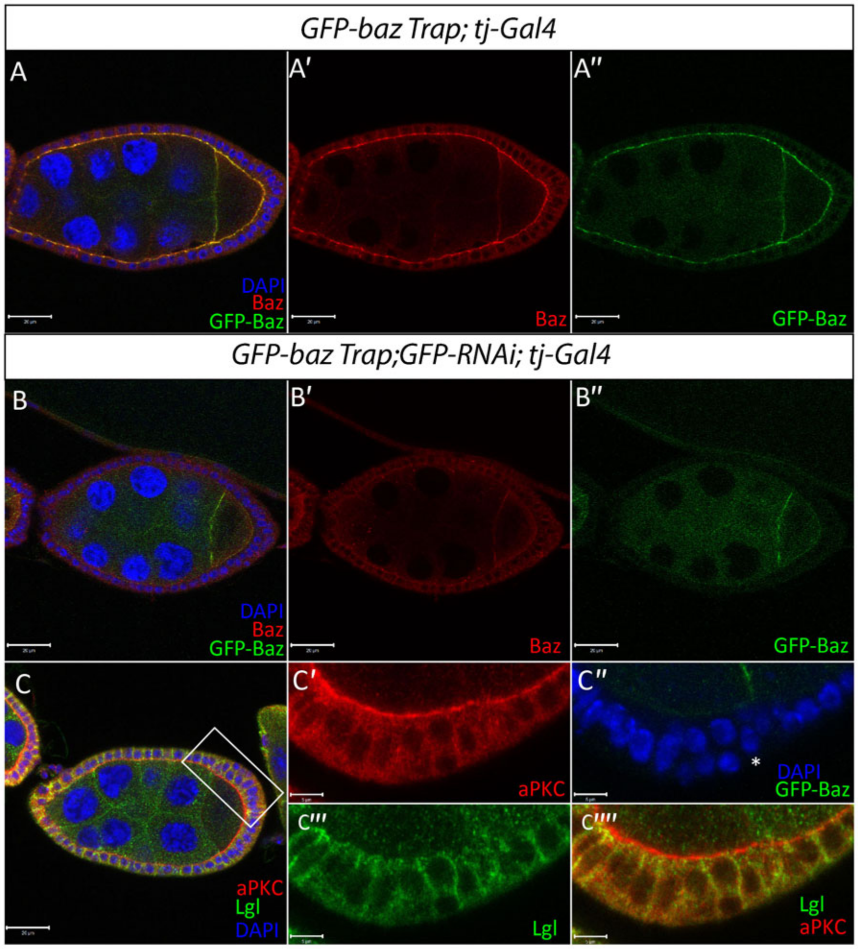

Fig. S2. RNAi knockdown of GFP-Bazooka in the FE phenocopies the *baz^{EH747}* phenotype. (A–A″) Stage 8 ovariole from *GFP-Baz Trap; tj-gal4* line immunostained with anti Baz N-term and GFP antibodies and stained with DAPI as indicated. (B–B″) Stage 7 ovariole from *GFP-Baz Trap; GFP-RNAi; tj-gal4* line immunostained with anti Baz N-term and GFP antibodies and stained with DAPI as indicated. Apical Baz is absent but FE integrity remains intact as visualized by uniform DAPI staining. (C–C″″) Stage 7 ovariole from *GFP-Baz Trap; GFP-RNAi; tj-gal4* line immunostained with aPKC, GFP and Lgl antibodies and stained with DAPI. Panels (C′–C″″) correspond to the boxed area in C. aPKC localizes to the apical membrane in lateral FE cells while PFCs display mild multilayering as indicated by the asterisk. Scale bars=20 µm in A–C; 5 µm in C′–C″″.

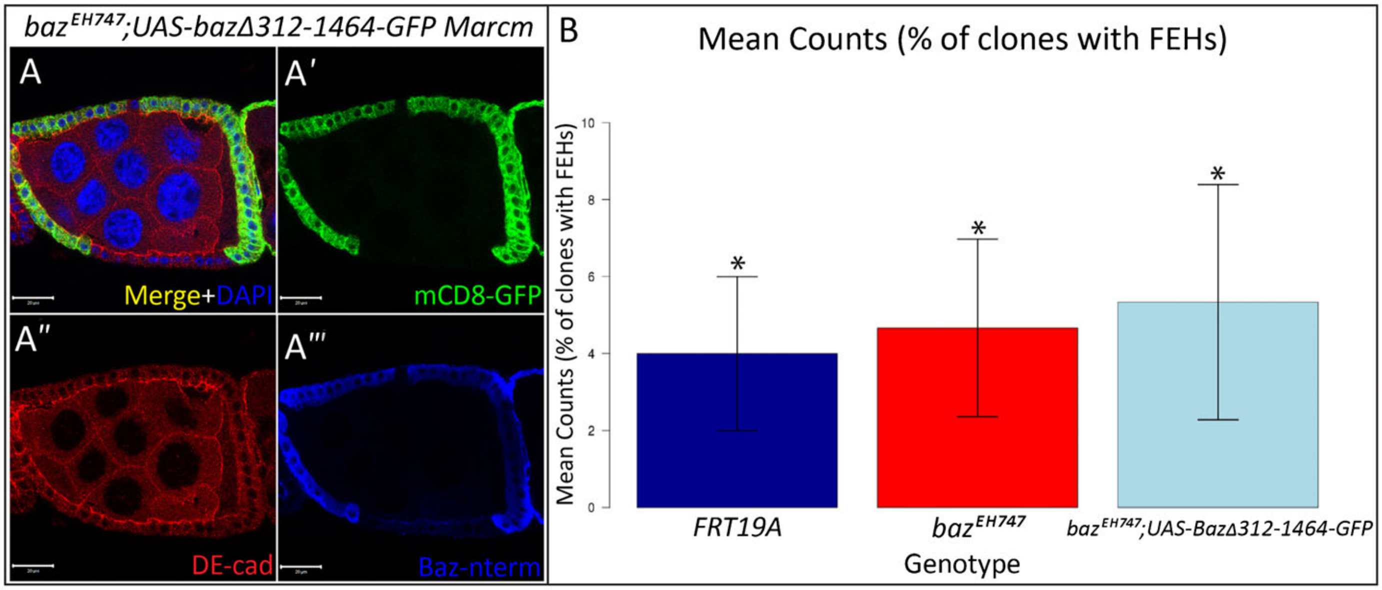

Fig. S3. Overexpression of a Baz N-terminal fragment does not cause FE defects. (A–A‴) Mosaic ovarian follicles in which *baz^{EH747} FRT19A*; *UAS-bazΔ312-1464-GFP* mutant follicle cells were generated using the MARCM technique by *hs-Flp* mediated recombination and marked by expression of mCD8-GFP. Follicles were immunostained with anti-DE-cad, anti-GFP and anti Baz N-term. A–A‴ shows a coronal section of a mosaic follicle in which the FE forms a continuous sheet over the nurse cells and oocyte as assessed by DE-cad immunostaining (A″) in spite of high levels of UAS-BazΔ312-1464 as confirmed by Baz N-term antibody staining (A‴). (B) Bar graph presenting quantitative analysis of the FEH phenotype. At $p<0.001$, genotypes with the same number of stars are not significantly different from each other. Scale bars=20 µm.

Biology Open (2015) 000, 1–14 doi:10.1242/bio.201410934

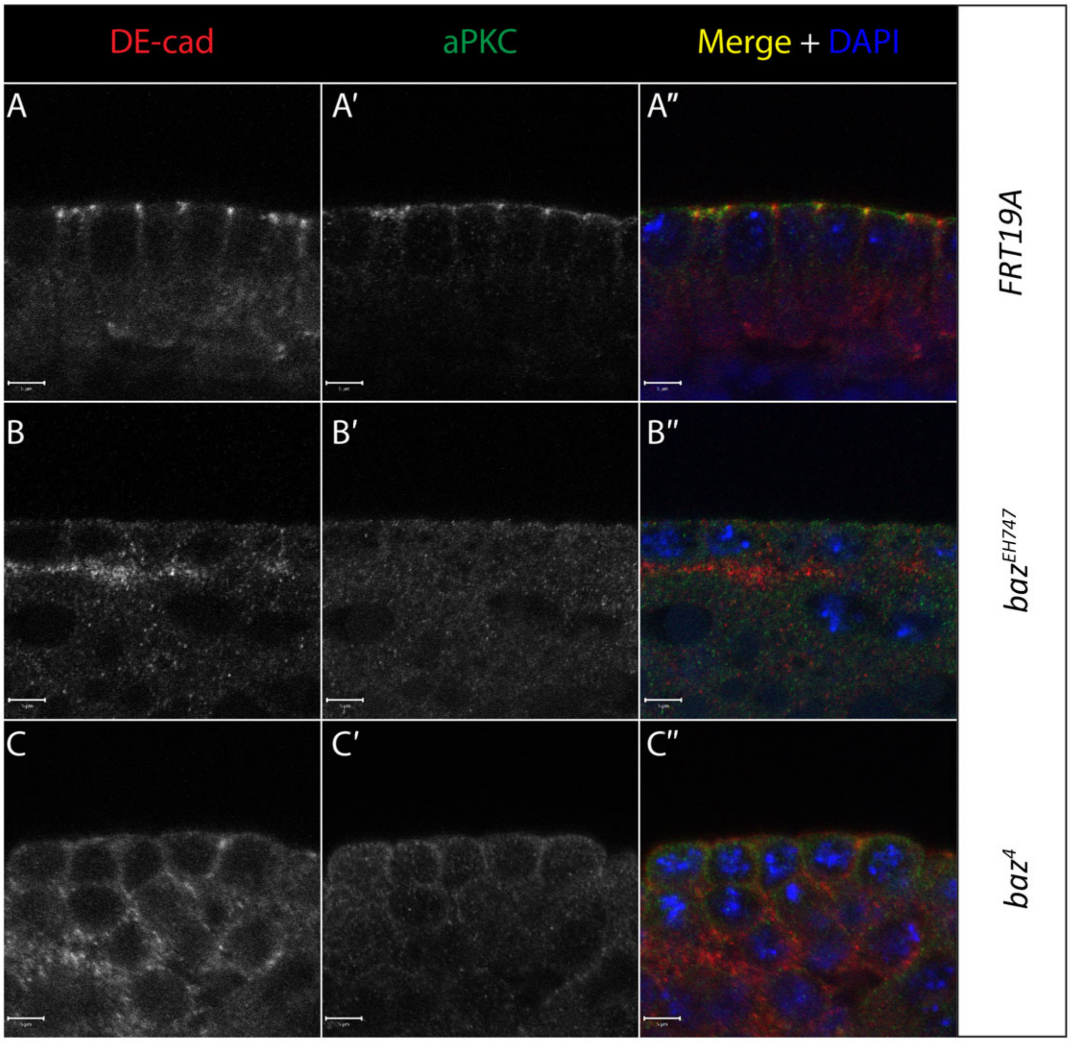

Fig. S4. Establishment of epithelial polarity is compromised in *baz*^{4} and *baz*^{EH747} maternal zygotic embryos. *FRT19A* control (A–A″), *baz*^{EH747} (B–B″) and *baz*^{4} (C–C″) stage 5 maternal zygotic mutant embryos were immunostained with antibodies against DE-cad and aPKC and stained with DAPI. *FRT19A* stage 5 embryonic epithelia show apical localization of DE-cad and aPKC (A,A′). By contrast, neither DE-cad nor aPKC localize apically in embryonic stage 5 *baz*^{EH747} (B,B′) or *baz*^{4} (C′,C″) maternal zygotic mutant epithelia. Scale bars=5 µm.

Biology Open

Biology Open (2015) 000, 1–14 doi:10.1242/bio.201410934

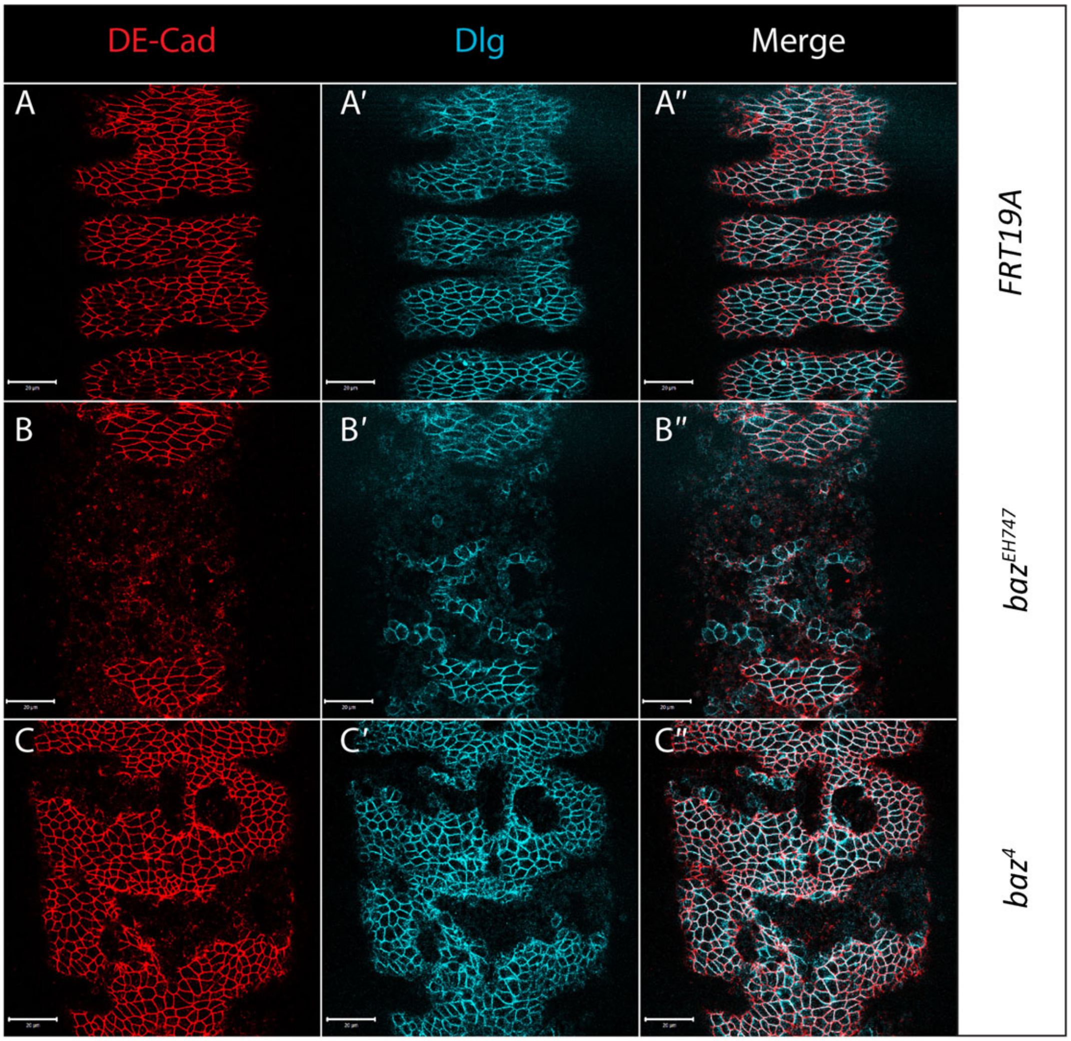

Fig. S5. Ventral epidermal integrity is severely disrupted in *baz*^{EH747} and *baz*^{4} zygotic mutant embryos. Ventral view of Stage 13 *FRT19A* control (A–A″), *baz*^{EH747} (B–B″) and *baz*^{4} (C–C″) male embryos immunostained with antibodies against DE-cad, Dlg and beta-galactosidase (not shown). *baz*^{EH747} and *baz*^{4} alleles were balanced over the *FM7 ftz-lacz* balancer chromosome and male embryos were identified by the absence of beta-galactosidase immunostaining. *FRT19A* (A–A″) embryos display a continuous epidermis as assessed by DE-cad (A) and Dlg (A′) immunostaining. *baz*^{EH747} (B–B″) and *baz*^{4} (C–C″) mutant embryos display large holes in the ventral epidermis as visualized by absence of DE-cad (B,C) and Dlg (B′ and C′) immunostaining. The gaps in the staining for DE-cad and Dlg of *FRT19A* embryos are caused by segmental folds in the epidermis that must not be mistaken for holes. Scale bars=20 µm.

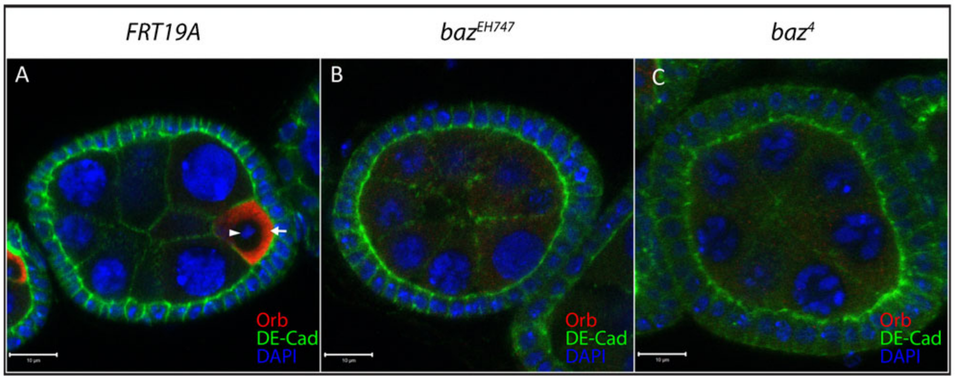

Fig. S6. Requirement for *baz* in oocyte development. Germline clones were generated using the *ovoD* dominant female sterile technique for *FRT19A* (A), *baz*^{EH747} (B) and *baz*^{4} (C), immunostained with antibodies raised against Orb and DE-cad and stained with DAPI as indicated. Stage 3 follicles carrying *baz*^{EH747} (B) and *baz*^{4} (C) germline clones often lack distinct Orb staining and contain only polyploid nuclei, indicating that they fail to maintain oocyte fate in comparison to *FRT19A* germline clones, which maintain polarized Orb staining (white arrow) and an oocyte with DNA compacted into a karyosome (white arrowhead). Scale bars=10 µm.

Biology Open

Biology Open (2015) 000, 1–14 doi:10.1242/bio.201410934

Table S1. Mutation of *baz* leads to loss of larval neuroblasts in 3rd instar larval brains

	Number of GFP positive Neuroblasts observed	Number of GFP positive Clones assessed	% of clones containing a GFP positive neuroblast
baz^{EH747}	0	37	0%
baz^{4}	0	39	0%
FRT19A	27	45	60%

Neuroblast MARCM clones for baz^{4}, baz^{EH747} and *FRT19A* were generated in 3rd instar larval brains. The number of clones containing a GFP positive neuroblast was assessed for each genotype. No GFP positive neuroblasts were observed in baz^{4} (0/37) or baz^{EH747} (0/39) clones while 60% of FRT19A clones (27/45) contained a GFP positive neuroblast.
